# Half-Dose Photodynamic Therapy as a Novel Treatment Protocol for Circumscribed Choroidal Hemangioma

**DOI:** 10.3390/life12111748

**Published:** 2022-10-31

**Authors:** David Pérez-González, Michaella Goldstein, Matias Iglicki, Dinah Zur

**Affiliations:** 1 Ophthalmology Division, Tel Aviv Medical Center, Sackler Faculty of Medicine, Tel Aviv University, Tel Aviv-Yafo 6234906, Israel; 2 Private Retina Practice, University of Buenos Aires, Buenos Aires 1414, Argentina

**Keywords:** choroidal hemangioma, choroidal tumor, photodynamic therapy, novel treatment

## Abstract

We present a case series of four patients with circumscribed choroidal hemangioma (CCH) treated with half-dose PDT, proposing this as a novel treatment protocol. Four patients with CCH were included, and then evaluated using multimodal imaging, including optical coherence tomography (OCT), fluorescein angiography, indocyanine green angiography, fundus autofluorescence, and ultrasound following treatment with half-dose and full-fluence PDT. Following half-dose PDT, all patients showed significant shrinkage of the hemangioma, functional improvement, and decrease of intra- and sub-retinal fluid. All patients remained stable after a single PDT treatment, with a follow-up of up to 60 months. No side effects were shown. This is the first report showing long term efficacy of half-dose PDT treatment in cases with CCH. The outcomes from this pilot study are comparable with results using full dose PDT protocols and it can be considered as a viable treatment option for CCH during the ongoing global verteporfin shortage.

## 1. Introduction

Circumscribed choroidal hemangioma (CCH) is a benign vascular tumor and comprises well-defined lesions, generally isolated at the posterior pole. Although it can be present from childhood, it is often asymptomatic until adulthood and can be found during routine examinations. CCH can cause metamorphopsia, visual loss, or visual field defects, due to serous retinal detachment, cystoid macular edema, or extravasation of intraretinal lipids [1]. Treatment is warranted when signs of exudation are present causing visual deterioration [1,2].

Due to the vascular characteristics of the hemangioma, photodynamic therapy (PDT) provides an effective treatment and is considered the gold standard therapeutic approach for this condition [1,3].

Since the introduction of PDT for CCH in 2003, ref. [4] several case reports and case series have shown high rates of anatomical tumor reduction with parallel visual improvement using the established laser parameters of full dose and full fluence of PDT modality [3,5].

One of the potential benefits of decreasing the verteporfin dosage might be reduced risk and/or severity of side effects, which have been reported previously when standard PDT protocol is applied [6,7,8,9,10,11]. In the setting of central serous chorioretinopathy (CSCR), half-fluence and/or half-dose verteporfin PDT have become the standard of treatment [12]. Lately, half-fluence PDT has been reported in a small cohort of patients with CCH [13].

As the world faces an ongoing shortage of verteporfin, reduced dose treatment might not only be beneficial for patient’s safety, but also make treatment available.

We present 4 consecutive cases of CCH that were treated with half dose and full fluence PDT, all of them exhibiting good anatomical and functional outcomes comparable with standardized PDT (full dose-full fluence). To the best of our knowledge, this is the first report that shows half-dose PDT modality as a viable treatment option for CCH.

## 2. Case Number 1

**Figure 1:**
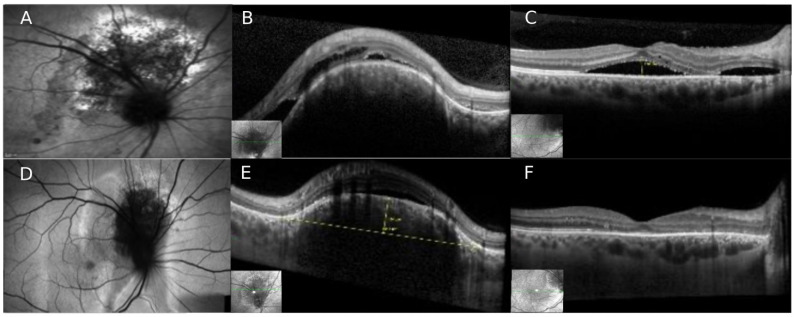
Circumscribed choroidal hemangioma treated with half-dose photodynamic therapy (Case nr. 1). (**A**) Fundus Autofluorescence (FAF) imaging shows the area of the peripapillary hemangioma superior to the optic disc. (**B**,**C**) Spectral-domain OCT (SD-OCT) imaging revealing a dome-shaped elevated choroidal lesion with smooth anterior surface and overlying RPE changes with sub- and intraretinal fluid that extended through the nasal portion reaching the macula. (**D**–**F**) After half-dose PDT, significant shrinkage of the CCH is seen on FAF and on OCT with complete resolution of subretinal fluid in the macular zone.

A 39-year-old healthy male was referred to our department with a previous diagnosis of central serous chorioretinopathy (CSCR). He complained of decreased vision and metamorphopsia in his right eye (OD) with further deterioration in the past weeks. Patient’s best corrected visual acuity (BCVA) was 20/40 OD and 20/25 in his left eye (OS). The anterior segment was normal in both eyes. Fundoscopic examination in OD revealed an elevated, oval, orange-red and well-defined lesion, 3-disc areas (DA) in size, located superior to the optic nerve (Figure 1). The macula was slightly elevated with dull foveal reflex and clinical evidence of subretinal fluid (SRF) in continuity with the area of the lesion superior to the disc. The fundoscopic examination on OS did not show any abnormality. Spectral domain OCT (SD-OCT) revealed a dome-shaped elevated choroidal lesion with smooth anterior surface, overlying retinal pigment epithelium (RPE) changes, and evidence of SRF and intraretinal fluid (IRF) that extended through the nasal portion of the macula, involving the foveal zone. Fundus autofluorescence (FAF) imaging revealed hypo-autofluorescence with some alternating areas of hyper-autofluorescence, all within the tumor zone. Fluorescein angiography (FA) showed mild hyper-fluorescence in the early arterial phase with diffuse intense hyper-fluorescence in the late phases. Indocyanine green angiography (ICGA) exhibited early hyper-fluorescence during the first minute, with the typical “washout” phenomenon in the late phases (after 20 min). Ultrasound confirmed the diagnosis of CCH, showing an acoustically solid mass with high internal hyper-echogenicity, measuring 7.2 mm in diameter and 2.9 mm height.

Due to the large amount of fluid, the patient was referred for combined therapy: After adjuvant treatment [13] of two monthly intravitreal bevacizumab injections (1.25 mg, Avastin, Genentech, San Francisco, CA, USA), only mild decrease of SRF and IRF was noted, and the CCH appeared unchanged. One month after the second intravitreal injection, half-dose full-fluence PDT was performed. Intravenous verteporfin was administered at a dosage of 3 mg/m^2^. 15 min after the beginning of intravenous infusion, 2 spots diode laser (689 nm) of 5200 nm^2^ and 4400 nm^2^ at full fluence (600 mW/cm^2^) and light dose of 59 J/cm^2^ were applied for 83 s.

3 months after the PDT session, the tumor had shrinked significantly to a height of 1.0 mm. OCT showed complete resolution of all retinal fluid (Figure 1), and BCVA improved to 20/25. During follow up of 5 years after PDT the patient remained stable; during the last follow-up only an isolated small amount of SRF nasal to the fovea and in the area of the hemangioma remained with no significant visual changes.

## 3. Case Number 2

**Figure 2:**
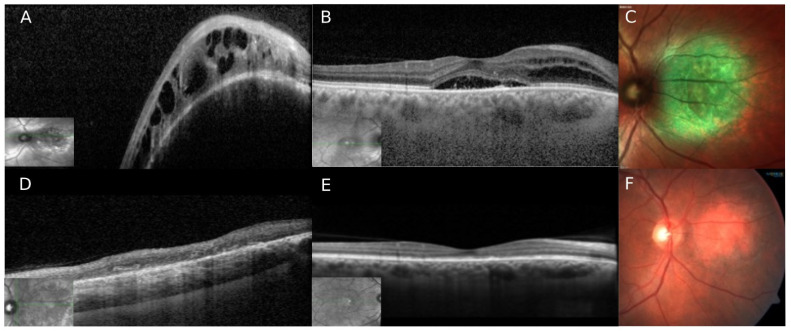
Circumscribed choroidal hemangioma treated with half-dose photodynamic therapy (Case nr. 2). (**A**,**B**) Spectral Domain OCT (SD-OCT) revealed a dome-shaped elevated choroidal lesion nasal to the disc with a smooth anterior surface, overlying RPE changes, and evidence of sub- and intraretinal fluid. The macular area shows an elevated contour with a sub-foveal pocket of SRF, and several hyper-reflective dots and RPE changes are seen in the same area. Nasal to the fovea, a small amount of IF is seen. (**C**) Multicolor image showing the hemangioma nasal to the optic disc. (**D**,**E**) After half-dose PDT, complete shrinkage of the tumor is seen with fluid reabsorption in the macula. (**F**) Color fundus picture of the tumor area showing tumor resolution with mild pigmentary changes.

A 49-year-old male was referred to our department due to an elevated peripapillary mass on his OD, diagnosed on an ophthalmic routine check-up. He was recently diagnosed with Diabetes Mellitus type 2 and denied any past ocular history. BCVA was 20/60 OD and 20/25 OS. Anterior segments were normal. OD fundoscopy revealed an elevated, round, orange-red, and well-defined lesion of approximately 5DA in size located nasal to the optic disc. Overlying the tumor area there was evidence of SRF extending to the macula and involving the fovea. The funduscopic examination of OS was normal, and without evidence of diabetic retinopathy. SD-OCT revealed an elevated choroidal dome-shaped lesion, with IRF and SRF extending to the macula and the fovea (Figure 2). FAF revealed hypo-autofluorescence with some alternating areas of hyper-autofluorescence, mainly seen in the area of the hemangioma. FA and ICGA showed typical signs of CCH. On ultrasound, the lesion appeared as a hyper-echogenic choroidal lesion with high internal reflectivity, measuring 1.7 mm in height and 6 mm in diameter. After establishing the diagnosis of CCH with macular involvement and decrease in vision, the patient underwent half-dose full-fluence PDT, following the protocol described previously. One spot (6500 µm^2^) was applied to cover the whole area of the hemangioma. 2 months after therapy, complete resolution of IRF and SRF in both, the hemangioma and macular zone area was appreciated, with significant reduction of the tumor size (Figure 2). BCVA improved from 20/50 to 20/30. The patient remained stable over a follow up period of 15 months.

## 4. Case Number 3

A 44-year-old male experienced decreased vision in OD since the past 4 years. He was initially diagnosed with CSCR in a community clinic and treated with 3 bevacizumab injections, without improvement. After revision of the clinical findings, he was diagnosed with CCH.

**Figure 3:**
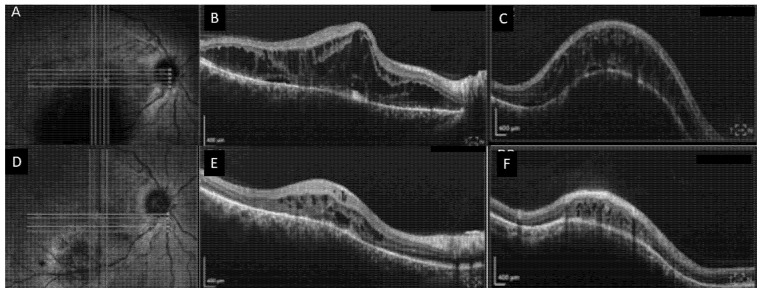
Circumscribed choroidal hemangioma treated with half-dose photodynamic therapy (Case nr. 3). (**A**) Near-infrared image showing an elevated choroidal lesion inferior to the fovea in the right eye. (**B**,**C**) Corresponding horizontal (**B**) spectral domain OCT (SD-OCT) scan through the fovea showing diffuse retinal cystoid changes and little subretinal fluid. Subfoveally, RPE irregularity can be noted. Corresponding vertical (**C**) SD-OCT scan revealed a dome-shaped elevated choroidal lesion inferior to the fovea with a smooth anterior surface, overlying RPE changes, and evidence of sub- and intraretinal fluid. (**D**) 8 weeks after half-dose PDT, shrinkage of the hemangioma is seen on the near-infrared image. (**E**) Horizontal SD-OCT scan showing complete resolution of the subretinal fluid with a residual amount of intraretinal fluid, and (**F**) vertical scan showing significant shrinkage of the CCH with improvement in retinal thickness.

BCVA was 20/400 OD and 20/20 OS. Anterior segments were normal. Fundoscopic examination in the OD revealed an elevated red-orange lesion of approximately 6DA, located in the inferior macula and overlying retinal fluid extending to the fovea. SD-OCT showed a significant amount of IRF with isolated pockets of SRF extending from the enlarged choroidal hemangioma inferiorly to the macula (Figure 3). ICGA showed typical signs of CCH. Ultrasound showed an acoustically solid choroidal mass with high internal reflectivity, measuring 3.74 mm in height and 7.72 mm in diameter.

The patient underwent half-dose full-fluence PDT, using one large spot of 6700 µm^2^ covering the whole area of the hemangioma. 8 weeks after treatment, significant anatomical improvement was appreciated with a residual amount of intraretinal fluid adjacent to the CCH, and shrinkage of the hemangioma to a height of 1.6 mm. BCVA improved from 20/400 to 20/100. The patient remained stable over a follow-up of 28 months.

## 5. Case Number 4

**Figure 4:**
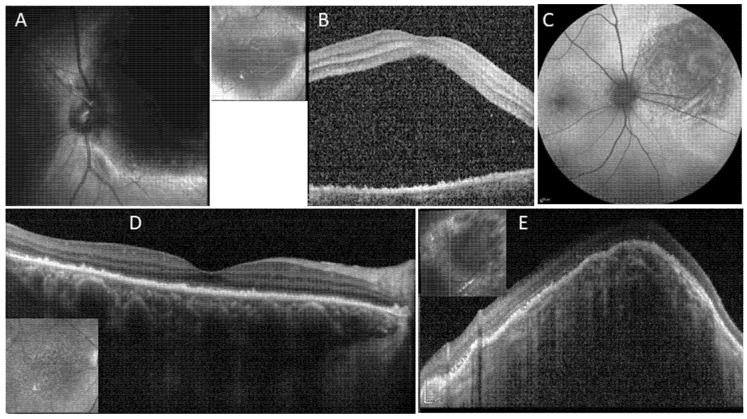
Circumscribed choroidal hemangioma treated with half-dose photodynamic therapy (Case nr. 4). (**A**,**B**) Near-infrared image showing the elevated large choroidal lesion nasal to the optic disc in the right eye. Spectral Domain OCT (SD-OCT) revealed a large amount of subretinal fluid. Additionally, RPE changes (**C**). After half-dose PDT, fundus autofluorescence imaging shows macular irregularity and nasal to the disc, hypo-autofluorescence in the treated area. (**D**) 8 weeks after treatment, complete resolution of all subretinal fluid on SD-OCT. (**E**) Multicolor image showing the hemangioma nasal to the optic disc. (**E**) The CCH, which was too high to be displayed on SD-OCT before treatment, has shrinked remarkably, irregularity of the overlying RPE is seen.

A 72-year-old female presented with gradual decrease in vision in her right eye. She was healthy without past ocular history. BCVA was 20/100 OD and 20/25 OS. In BE there was normal anterior segment with mild nuclear sclerosis of the lens, and fundus exam revealed early AMD on OS. Fundoscopic examination of her OD showed an elevated, large red-orange lesion of approximately 8DA located nasal to the optic disc, and SRF and IRF extending to the macula involving the fovea. On SD-OCT, the lesion appeared as a choroidal dome-shaped elevation with overlying SRF and IRF, extending to the macula and fovea in a large amount (Figure 4). Foveal RPE changes were present as well. FA and ICGA showed typical characteristics of CCH. On ultrasound, the lesion measured 7.9 mm in diameter and 5.3 mm in height, appearing as a solid dome-shaped mass with medium to high internal reflectivity. The patient underwent half dose PDT applying three large spots of 7900 µm^2^, covering the area of the hemangioma and sparing the optic disc. Eight weeks after treatment, the patient showed significant improvement in vision with a BCVA of 20/30 OD. Anatomically, a remarkable tumor reduction was seen with re-absorption of all SRF and IRF. At last follow-up 12 months after PDT the patient remained stable.

## 6. Discussion

To our best knowledge, this is the first case series of patients with CCH treated by application of a half-dose PDT protocol. We achieved good anatomical success in terms of tumor shrinkage as well as fluid resolution with consequent improvement in visual acuity.

Full-fluence full-dose PDT has been in use for CCH since 2003, and its effectiveness was first demonstrated in a prospective study by Jurklies et al., achieving reduction of exudation in 94.8% of cases and reduction in tumor size in all included patients, while VA improved by at least one line in 73.3% cases, and by two lines in 42.1% [4]. Compared to radiotherapy, PDT have showed a better safety profile with lower complication rates, while tumor reduction and visual acuity outcomes were comparable in both modalities [11].

The study that put the seal for PDT as gold standard in treating CCH was published by Shields et al. in 2019 [14]. In this comprehensive analysis, 452 cases over 51 years were included, and then a comparison was made between treatments in the pre-PDT and post-PDT eras. Those who received PDT bestowed a better visual prognosis with a mean VA of 20/63, compared to 20/400 of those treated with other modalities in the pre-PDT era such as radiotherapy and laser photocoagulation. These findings were particularly significant in patients whose initial visual acuity was equal to or better than 20/200. On the other hand, tumor size reduction in conjunction with a more controlled cystoid macular edema was also remarked in patients treated with PDT. It is important to note that this study is not analyzing specific therapies but different frame times with varying treatment resources. This demonstrates that progress in visual acuity was achieved after PDT was incorporated for treating CCH [14].

The exact mechanism of how PDT causes tumor shrinkage is not well understood. Prior studies on its use for neovascular membranes imply a selective vascular occlusion after verteporfin infusion and dye stimulation [15,16,17]. Although CCH has a standard endothelial lining, it is speculated that the localized effect of PDT could be associated with slower perfusion characteristics in these tumors with a similar molecular expression of low-density lipoproteins as identical with vascular proliferation in other pathologies [11,14].

Despite the apparent benefit of PDT, side effects were described; such as phototoxicity, choroidal ischemia, retinal vascular occlusion, neovascularization, polypoidal vascular formation, subretinal hemorrhage, choroidal atrophy, and neuro-sensory retinal degeneration [3,11,13]; all of these can lead to significant visual impairment [3,11,13]. Hence, decreasing the rate of complications is desirable. In prior CCH studies, patients were treated by PDT in a classic full-dose modality [1,3,5]. Modified approaches using half-dose and/or half-fluence modalities have been evaluated in treating CSCR and polypoidal choroidal vasculopathy, with non-inferior anatomical and functional outcomes vs. standard PDT treatment protocols [12,18,19]. In the current study, we achieved tumor shrinkage and fluid resolution in all cases using our half-dose PDT algorithm. No side effects were present.

In addition to the potentially improved safety profile, the VA and anatomical results, highly suggest that this treatment modality can be widely adopted. Since May 2020, it has been a marked worldwide lack of verteporfin supply; this is attributed to a reduction in manufacturing capacity, with uncertainty remaining in the short-term future [20]. In this sense, our technique might offer a viable approach during this era of shortage in addition to safety considerations.

Previous studies showed that cytotoxicity and vascular damage related to verteporfin are dose-dependent [10,21,22].

In conclusion, based on our pilot study, half-dose PDT offers a safe and efficient treatment option for CCH, leading to tumor shrinkage, fluid resorption and improvement in visual acuity. Larger studies will be needed to define the role of half-dose PDT in the current treatment algorithm of CCH.
